# The Influence of 24-h Ambulatory Blood Pressure on Cognitive Function and Neuropathological Biomarker in Patients With Alzheimer's Disease

**DOI:** 10.3389/fnagi.2022.909582

**Published:** 2022-06-22

**Authors:** Lixia Li, Weijia Wang, Tenghong Lian, Peng Guo, Mingyue He, Weijiao Zhang, Jinghui Li, Huiying Guan, Dongmei Luo, Weijia Zhang, Wei Zhang

**Affiliations:** ^1^Department of Internal Medicine in International Medical Services, Beijing Tiantan Hospital, Capital Medical University, Beijing, China; ^2^Center for Cognitive Neurology, Department of Neurology, Beijing Tiantan Hospital, Capital Medical University, Beijing, China; ^3^Department of Neurology, Beijing Tiantan Hospital, Capital Medical University, Beijing, China; ^4^China National Clinical Research Center for Neurological Diseases, Beijing, China; ^5^Beijing Institute for Brain Disorders, Capital Medical University, Beijing, China; ^6^Beijing Key Laboratory on Parkinson's Disease, Beijing, China

**Keywords:** Alzheimer's disease, mild cognitive impairment, dementia, 24-h ambulatory blood pressure, cognitive domains, neuropathological biomarkers of AD

## Abstract

**Purpose:**

This study aimed to investigate the influence of 24-h ambulatory blood pressure (BP) on cognitive function and neuropathological biomarkers in patients with Alzheimer's disease (AD) at the stages of mild cognitive impairment (MCI) and dementia.

**Methods:**

The patients with AD were divided into the MCI (AD-MCI) group and the dementia (AD-D) group. Notably, 24-h BP variables, including BP level, coefficient of variation (CV) of BP, and pulse pressure, were collected and compared between the two groups. The correlations between 24-h BP variables and the scores of cognitive domains were analyzed. The independent influencing factors of cognitive domains of patients with AD were investigated. The levels of neuropathological biomarkers of AD, including β amyloid (Aβ)_1−42_, phosphorylated tau (P-tau), and total tau (T-tau), in cerebrospinal fluid (CSF) were measured and compared between the two groups, and the correlations between 24-h BP variables and the levels of neuropathological biomarkers of AD were analyzed.

**Results:**

Daytime CV of systolic BP (SBP) was significantly increased in the AD-D group compared to that in the AD-MCI group. The 24-h and daytime CV of SBP and ambulatory pulse pressure were significantly and negatively correlated with memory score. The average 24-h and average daytime SBP level and CV of SBP, daytime CV of diastolic BP (DBP), and 24-h, daytime, and night-time ambulatory pulse pressure were significantly and negatively correlated with language score. The average 24-h SBP level, daytime CV of SBP, and 24-h, daytime, and night-time ambulatory pulse pressure were significantly and negatively correlated with attention score. Further analysis indicated that daytime CV of SBP as well as age and course of disease were the independent influencing factors of language. Age was also the independent influencing factor of memory and attention of patients with AD. T-tau level in CSF in the AD-D group was significantly higher than that in the AD-MCI group, but the levels of Aβ_1−42_, P-tau, and T-tau in CSF were not correlated with 24-h ambulatory BP variables.

**Conclusion:**

Daytime CV of SBP was the independent influencing factor of language in patients with AD. The AD-D patients had significantly severe neurodegeneration than AD-MCI patients, which was, however, not through the influence of 24-h ambulatory BP variables on neuropathological biomarkers of AD.

## Introduction

Alzheimer's disease (AD) is the most common neurodegenerative disease and a type of dementia in the elderly. With the accelerated aging process, its incidence and prevalence are increasing year after year. The main clinical manifestations of AD, which is characterized by a progressive deterioration in cognition, behavior, and function, place a considerable burden on society. AD can be conceptualized as a continuum, i.e., patients progress from normal cognition to mild cognitive impairment (MCI) due to AD (AD-MCI), followed by the increased severity of dementia due to AD (AD-D) (Davis et al., 2018).

Abnormal blood pressure (BP) was closely related to the occurrence and development of AD. The relationship between BP level and cognitive function in patients with AD was complex, which was reflected by the evidence that either high or low BP level might increase the risk of cognitive impairment and dementia (Streit et al., 2019). BP variability (BPV) refers to the fluctuation degree of BP in a specific time, and its abnormal increase reflects the increase of BP fluctuation degree. It was found that elevated BPV was a predictive of dementia, independent of the average BP level (Sible and Nation, 2021b). However, other studies demonstrated that elevated BPV was not associated with an increased risk of dementia (van Middelaar et al., 2018). Pulse pressure is calculated as the systolic BP (SBP) values minus diastolic BP (DBP) values, and a previous study showed that it was a potential key contributor to cognitive impairment in many individuals (Thorin-Trescases et al., 2018). Previous studies on BP and AD have mainly focused on the relationship between the occurrence of AD and office BP level, BPV, and pulse pressure, and have rarely investigated the differences of 24-h ambulatory BP variables in patients with AD at the stages of MCI and dementia and the influence of 24-h ambulatory BP variables on multiple cognitive domains of patients with AD. The relationship between 24-h ambulatory BP variables (e.g., BP level, BPV, and pulse pressure) and cognitive function in patients with AD remains uncertain.

The neuropathological features of AD include the neuritic plaques and the neurofibrillary tangles, which were mainly composed of β amyloid (Aβ) and phosphorylated tau (P-tau), respectively. The levels of the neuropathological biomarkers of AD in cerebrospinal fluid (CSF) were closely related to the degree of cognitive impairment in patients with AD (Milà-Alomà et al., 2019; Bridel et al., 2022). It was previously found that BPV was related to increased tau accumulation over time specifically within a temporal region known to show tau deposition on a tau-PET scan during the early stages of AD (Sible and Nation, 2021b). However, it is still unclear whether BP variables affect the disease severity and impairments of cognitive domains by changing the level of pathological biomarkers in CSF in patients with AD.

In this study, we compared 24-h ambulatory BP variables (e.g., BP level, BPV, and pulse pressure) between AD-MCI and AD-D groups, analyzed the correlations of 24-h ambulatory BP variables with the score of multiple cognitive domains, and identified the independent factor that influenced individual cognitive domains. We measured and compared the levels of neuropathological proteins in CSF between AD-MCI and AD-D groups, and further analyzed the correlation between the levels of neuropathological biomarkers in CSF and BP variables in patients with AD.

## Materials and Methods

### Ethics Statement

This study met the guidelines of ethical principles for medical research involving human subjects of the Declaration of Helsinki, and the study protocol was approved by the Ethical Review Board of Beijing Tiantan Hospital. Written informed consents were obtained from patients with AD and their family members. All methods were performed following relevant guidelines and regulations.

### Inclusion Criteria of AD

This study included patients with MCI due to AD (Albert et al., 2011) and patients with AD dementia (McKhann et al., 2011) according to the National Institute of Aging and Alzheimer's Association (NIA-AA) criteria (Albert et al., 2011; McKhann et al., 2011).

### Exclusion Criteria for Enrolled Patients With AD

- The MCI and dementia caused by the following diseases were excluded: (1) Parkinson's syndrome with early significant visual hallucinations and rapid eye movement sleep behavior disorder, which was common in Lewy body disease; (2) multiple vascular risk factors and/or head MRI suggested extensive cerebrovascular disease, which might suggest vascular cognitive impairment; (3) significant behavioral or language impairment early in the course of disease, which might suggest frontotemporal lobar degeneration; (4) rapid cognitive decline within a few weeks or months might indicate prion disease, tumor, or metabolic disease; and (5) other diseases were excluded, which might cause cognitive decline.
- The patients who could not cooperate with ambulatory BP monitoring and cognitive evaluation by a variety of rating scales due to various reasons were excluded.
- The patients with atrial fibrillation, atrial flutter, acute myocardial infarction, myocarditis, and severe valvular heart disease were excluded.

### Collections of Demographic Variables and Clinical Information

A total of 190 hospitalized patients with AD according to the inclusion and exclusion criteria were consecutively recruited from the inpatient wards of the Departments of Neurology and Geriatrics, Beijing Tiantan Hospital, Capital Medical University, from November 2017 to November 2021.

The general demographic and clinical data of patients with AD, including gender, age, course of disease, body mass index (BMI), and education level, were recorded; the number of patients with smoking history, alcohol history, hypertension, diabetes, and coronary heart disease, and the number of patients taking antihypertensive medicines were also recorded.

### Ambulatory BP Monitoring

A monitor (model MOBIL-O-GRAPH) made in Germany was used to observe 24-h ambulatory BP for each patient with AD before adjustment of antihypertensive medicines within 3 days after admission.

On the day of ambulatory BP monitoring, the patients with AD worked and rested according to the daytime and night-time, continued taking drugs according to the previous medication situation, and kept the activities of daily living unaffected. BP was recorded every 30 min during the day (6 a.m.−10 p.m.), and every 60 min at night (10 p.m.−6 a.m.). If the record time of 24-h ambulatory BP monitoring was <22 h or the invalid record was >20% of the total record of BP, patients were eliminated or monitored 24-h ambulatory BP again. In this study, the coefficient of variation (CV) of BP over a specific period was used to reflect the BPV.

The average SBP and DBP in the whole day, day and night, and the CV of SBP and DBP in the corresponding period were collected, respectively. The ambulatory pulse pressure throughout the whole day, day, and night was collected, respectively.

### Assessment of Cognitive Function

The cognitive domains of patients with AD were assessed by using the following rating scales within 3 days after admission.

Memory: Auditory Verbal Learning Test (AVLT) (Guo et al., 2009) was used to assess verbal memory. AVLT1-3, AVLT4, and AVLT5 standed for immediate recall, short delayed recall, and long-delayed recall, respectively. The first 5 times of total recall of AVLT represented the general situation of verbal memory.

Language: Animal Fluency Test (AFT) (Zhao et al., 2018) was used to assess language function. The lower the score, the poorer the language function.

Attention: Symbol Digit Modalities Test (SDMT) (Zhou et al., 2019) was used to assess attention. The lower the score, the worse the attention.

### Measurements of Neuropathological Biomarkers of AD in CSF

Anti-AD drugs were withheld for 12–14 h prior to sampling the CSF. The volume of 3 ml CSF was obtained using a lumbar puncture between 7 a.m. and 10 a.m. under a fasting condition, and placed in a polypropylene tube. Approximately, the volume of 0.5 ml CSF was aliquoted into separate Nunc cryotubes and kept frozen at −80°C until used in assays.

The levels of neuropathological biomarkers of AD, including Aβ_1−42_, P-tau, and total tau (T-tau) in CSF, were detected by enzyme linked immuno sorbent assay (ELISA) with the kits of Immunobiological Laboratories Co., Ltd. (Japan).

### Statistical Processing

Statistical analysis was performed using the SPSS version 21.0 software. A value of *P* <0.05 was defined as statistically significant.

The demographic data and BP variables, including the average BP level, CV of BP and pulse pressure during 24-h monitoring, and neuropathological biomarkers in CSF from the AD-MCI and AD-D groups, were compared. If the data of two groups satisfied the normal distribution or the uniform variance, the deviation of the mean standard was performed to represent the comparison between the two groups by the *t*-test; if the data did not satisfy the normal distribution or the uniform variance, the data were presented by the medians (quartiles), while the comparison between the two groups adopted a nonparametric test. The categorical data were expressed as a percentage, and the χ^2^ test was used for comparison between the two groups.

The Spearman correlation was used to analyze the relationship between BP variables during 24-h monitoring and the rating scores of multiple cognitive domains, as well as neuropathological biomarkers in CSF from patients with AD.

The demographic data and BP variables during 24-h monitoring that might affect the rating scores of cognitive domains in patients with AD were further analyzed by multiple linear regression to reveal the independent factors that influenced each cognitive domain of patients with AD.

## Results

### Comparisons of Demographic Variables Between the AD-MCI and AD-D Groups

Among the 190 patients enrolled, 60 cases with the severity of MCI were in the AD-MCI group, and the remaining 130 cases with the severity of dementia were in the AD-D group.

The age in the AD-D group was significantly higher than that in the AD-MCI group (*P* <0.05). There was no significant difference in gender, course of disease, education level, BMI, smoking history, alcohol history, hypertension, diabetes, coronary heart disease, and the number of patients taking antihypertensive medicines between the two groups (Table 1).

**Table 1:** Comparisons of demographic variables between the AD-MCI and AD-D groups.

	**AD-MCI group (*n* = 60)**	**AD-D group (*n* = 130)**	***P-*value**
Male [*x*/*n* (%)]	34/60 (56.67%)	60/130 (46.15%)	0.178
Age (year, $\bar{X}\;\pm \;S$)	62.17 ± 8.82	67.38 ± 9.52	<0.001
Course of disease [month, M (Q1-Q3)]	24.00 (12.00–36.00)	36.00 (16.50–49.00)	0.088
Education level
Primary school and below [*x*/*n* (%)]	11/60 (18.33%)	21/130 (16.15%)	0.709
Secondary school [*x*/*n* (%)]	19/60 (31.67%)	54/130 (41.54%)	0.317
College and above [*x*/*n* (%)]	30/60 (50.00%)	55/130 (42.31%)	0.160
BMI [kg/m^2^, $\bar{X}\;\pm \;S$]	24.12 ± 2.96	23.73 ± 3.28	0.404
Smoking history [*x*/*n* (%)]	18/60 (30.00%)	27/130 (20.77%)	0.164
Alcohol history [*x*/*n* (%)]	20/60 (33.33%)	34/130 (26.15%)	0.308
Hypertension [*x*/*n* (%)]	24/60 (40.00%)	54/130 (41.54%)	0.821
Taking antihypertensive medicines [*x*/*n* (%)]	21/60 (35.00%)	47/130 (36.15%)	0.877
Diabetes [*x*/*n* (%)]	13/60 (21.67%)	28/130 (21.54%)	0.995
Coronary heart disease [*x*/*n* (%)]	4/60 (6.67%)	10/130 (7.69%)	0.801

*AD, Alzheimer's disease; AD-MCI, mild cognitive impairment due to AD; AD-D, dementia due to AD; BMI, body mass index*.

### Comparisons of 24-h Ambulatory BP Variables Between the AD-MCI and AD-D Groups

The 24-h ambulatory BP variables, including the average BP level, CV of BP, and pulse pressure in the AD-MCI and AD-D groups, were compared. It was shown that daytime CV of SBP in the AD-D group was significantly higher than that in the AD-MCI group. There were no significant differences in 24-h and night-time CV of SBP, CV of DBP in each period, average BP level, and pulse pressure between the AD-MCI and AD-D groups (Table 2).

**Table 2:** Comparisons of 24-h ambulatory BP variables between the AD-MCI and AD-D groups.

	**AD-MCI group (*n* = 60)**	**AD-D group (*n* = 130)**	***P-*value**
Average 24-h SBP level [mmHg, $\bar{\;X}\;\pm \;S$]	122.38 ± 15.28	122.98 ± 12.68	0.779
Average daytime SBP level [mmHg, $\bar{\;X}\;\pm \;S$]	123.47 ± 15.18	123.92 ± 13.31	0.837
Average night-time SBP level [mmHg, $\bar{\;X}\;\pm \;S$]	119.48 ± 16.80	120.55 ± 14.86	0.659
Average 24-h DBP level [mmHg, $\bar{\;X}\;\pm \;S$]	77.42 ± 9.04	76.95 ± 9.57	0.753
Average daytime DBP level [mmHg, $\bar{\;X}\;\pm \;S$]	78.53 ± 9.08	77.92 ± 9.85	0.681
Average night-time DBP level [mmHg, $\bar{\;X}\;\pm \;S$]	74.62 ± 10.40	74.73 ± 10.49	0.944
24-h CV of SBP [mmHg, M (Q1-Q3)]	11.90 (10.43–15.05)	13.40 (10.40–16.90)	0.112
Daytime CV of SBP [mmHg, M (Q1-Q3)]	11.35 (9.15–13.95)	12.50 (10.60–16.53)	0.034
Night-time CV of SBP [mmHg, M (Q1-Q3)]	9.90 (7.53–12.60)	10.05 (7.05–12.97)	0.391
24-h CV of DBP [mmHg, M (Q1-Q3)]	9.30 (8.20–11.98)	9.80 (8.05–11.45)	0.826
Daytime CV of DBP [mmHg, M (Q1-Q3)]	8.30 (7.33–11.80)	9.30 (7.45–11.30)	0.503
Night-time CV of DBP [mmHg, M (Q1-Q3)]	7.90 (6.13–9.77)	8.05 (6.00–9.95)	0.580
24-h ambulatory pulse pressure [mmHg, M (Q1-Q3)]	43.00 (38.00–51.00)	46.00 (40.00–51.00)	0.224
Daytime ambulatory pulse pressure [mmHg, M (Q1-Q3)]	42.00 (38.00–50.00)	46.00 (39.50–52.00)	0.240
Night-time ambulatory pulse pressure [mmHg, M (Q1-Q3)]	43.50 (37.00–52.00)	45.00 (39.00–52.00)	0.424

*AD, Alzheimer's disease; AD-MCI, mild cognitive impairment due to AD; AD-D, dementia due to AD; BP, blood pressure; SBP, systolic blood pressure; DBP, diastolic blood pressure; CV, coefficient of variation*.

### The Correlation Between 24-h Ambulatory BP Variables and the Scores of Multiple Cognitive Domains in Patients With AD

The bivariate Spearman correlation analysis was used to investigate the correlations between 24-h BP variables, including the BP level, CV of BP and pulse pressure, and the scores of rating scales for multiple cognitive domains, including memory, language, and attention in patients with AD (Table 3).

**Table 3:** The correlation analysis between 24-h ambulatory BP variables and the scores of multiple cognitive domains in patients with AD.

	**Memory (AVLT score)**		**Language (AFT score)**		**Attention (SDMT score)**	
	***R***	***P-*value**	***R***	***P-*value**	***R***	***P-*value**
Average 24-h SBP level	−0.148	0.055	−0.168	0.028	−0.169	0.049
Average daytime SBP level	−0.150	0.053	−0.172	0.025	−0.162	0.059
Average night-time SBP level	−0.065	0.403	−0.107	0.167	−0.132	0.126
Average 24-h DBP level	−0.078	0.313	−0.064	0.404	−0.044	0.607
Average daytime DBP level	−0.088	0.255	−0.060	0.437	−0.046	0.596
Average night-time DBP level	−0.020	0.795	−0.051	0.513	−0.018	0.835
24-h CV of SBP	−0.164	0.033	−0.177	0.021	−0.134	0.121
Daytime CV of SBP	−0.207	0.007	−0.249	0.001	−0.218	0.011
Night-time CV of SBP	0.116	0.136	0.094	0.221	0.091	0.291
24-h CV of DBP	−0.041	0.601	−0.125	0.105	0.031	0.717
Daytime CV of DBP	−0.057	0.462	−0.166	0.031	−0.017	0.841
Night-time CV of DBP	0.076	0.328	0.099	0.201	0.096	0.322
24-h ambulatory pulse pressure	−0.188	0.015	−0.198	0.010	−0.218	0.011
Daytime ambulatory pulse pressure	−0.196	0.011	−0.209	0.006	−0.213	0.013
Night-time ambulatory pulse pressure	−0.117	0.127	−0.153	0.046	−0.201	0.019

*BP, blood pressure; AD, Alzheimer's disease; AVLT, Auditory Verbal Learning Test; AFT, Animal Fluency Test; SDMT, Symbol Digit Modalities Test; SBP, systolic blood pressure; DBP, diastolic blood pressure; CV, coefficient of variation*.

It was found that 24-h and daytime CV of SBP and 24-h and daytime ambulatory pulse pressure were significantly and negatively correlated with AVLT score in patients with AD, indicating that the greater the abovementioned BP-related indexes, the more obvious the memory decline of patients with AD.

It was observed that the average 24-h and the average daytime SBP level, 24-h and daytime CV of SBP, daytime CV of DBP, and 24-h, daytime, and night-time ambulatory pulse pressure were significantly and negatively correlated with AFT score in patients with AD, suggesting that the greater the abovementioned BP-related indexes, the worse the language ability of patients with AD.

It was shown that the average 24-h SBP level, daytime CV of SBP, and 24-h, daytime, and night-time ambulatory pulse pressure were significantly and negatively correlated with SDMT score in patients with AD, implying that the greater the abovementioned BP-related indexes, the more obvious the attention disorder of patients with AD.

### Multivariate Regression Analysis of the Scores of Multiple Cognitive Domains in Patients With AD

Multiple linear regression analysis was conducted on the possible influencing factors affecting the AVLT score of patients with AD, including age, course of disease, the average 24-h SBP level, the average daytime SBP level, 24-h CV of SBP, daytime CV of SBP, 24-h ambulatory pulse pressure, and daytime ambulatory pulse pressure. The results showed that age was an independent factor affecting the memory of patients with AD, and the B-value was negative, suggesting that the older the age, the worse the memory of patients with AD (Table 4).

**Table 4:** Multivariate regression analysis of cognitive domain scores in patients with AD.

	**Memory (AVLT score)**		**Language (AFT score)**		**Attention (SDMT score)**	
	**B**	***P-*value**	**B**	***P-*value**	**B**	***P-*value**
Age	−0.400	<0.001	−0.138	0.019	−0.588	0.022
Course of disease	−0.040	0.114	−0.036	0.024	−0.022	0.702
Average 24-h SBP value	0.284	0.501	0.231	0.359	0.366	0.687
Average daytime SBP value	−0.425	0.306	−0.271	0.274	−0.600	0.499
24-h CV of SBP	0.652	0.241	0.516	0.116		
Daytime CV of SBP	−0.700	0.157	−0.676	0.034	−0.653	0.136
Daytime CV of DBP			0.082	0.694		
24-h ambulatory pulse pressure	−0.450	0.483	0.445	0.611	−1.341	0.642
Daytime ambulatory pulse pressure	0.620	0.327	−0.165	0.774	1.637	0.485
Night-time CV of SBP			−0.278	0.198	−0.166	0.885

*AD, Alzheimer's disease; AVLT, Auditory Verbal Learning Test; AFT, Animal Fluency Test; SDMT, Symbol Digit Modalities Test; B, regression coefficient or intercept; SBP, systolic blood pressure; DBP, diastolic blood pressure; CV, coefficient of variation*.

Multivariate linear regression analysis was adopted on the possible influencing factors affecting the AFT score of patients with AD, including age, course of disease, the average 24-h SBP level, the average daytime SBP level, 24-h CV of SBP, daytime CV of SBP, daytime CV of DBP, 24 h ambulatory pulse pressure, daytime ambulatory pulse pressure, and night-time ambulatory pulse pressure. The results showed that age, course of disease, and daytime CV of SBP were the independent influencing factors of language ability of patients with AD, and the B-values were negative, suggesting that the older the age, the longer the course of disease and the greater the variability of daytime SBP, the worse the language ability of patients with AD (Table 4).

Multivariate linear regression analysis was performed on the possible influencing factors affecting the attention of patients with AD, including age, course of disease, the average 24-h SBP level, the average daytime SBP level, daytime CV of SBP, 24-h ambulatory pulse pressure, daytime ambulatory pulse pressure, and night-time ambulatory pulse pressure. The results showed that age was an independent factor affecting the attention of patients with AD, and the B-value was negative, suggesting that the older the age, the worse the attention of patients with AD (Table 4).

### Comparisons of Neuropathological Biomarkers of AD Between the AD-MCI and AD-D Groups

Among the patients with AD enrolled in this study, 43 cases had CSF samples collected, including 12 patients in the AD-MCI group and 31 patients in the AD-D group. The levels of Aβ_1−42_, P-tau, and T-tau in CSF were measured and compared between the AD-MCI and AD-D groups. It was found that T-tau level in CSF from the AD-D group was significantly elevated compared with that from the AD-MCI group. There was no significant difference in the levels of Aβ_1−42_ and P-tau in CSF between the AD-MCI and AD-D groups (Table 5).

**Table 5:** Comparisons of neuropathological biomarkers of AD between the AD-MCI and AD-D groups.

	**AD-MCI group (*n* = 12)**	**AD-D group (*n* = 31)**	***P-*value**
Aβ_1−42_ [pg/ml, M (Q1-Q3)]	648.58 (403.84–2,075.58)	557.67 (343.58–765.45)	0.254
P-tau [pg/ml, M (Q1-Q3)]	50.38 (34.42–63.50)	53.66 (28.13–82.39)	0.883
T-tau [pg/ml, M (Q1-Q3)]	304.06 (132.35–553.61)	519.07 (365.16–771.53)	0.037

*AD, Alzheimer's disease; AD-MCI, mild cognitive impairment due to AD; AD-D, AD dementia; Aβ, β amyloid; P-tau, phosphorylated tau; T-tau, total tau*.

### Correlations Between Neuropathological Biomarkers of AD and 24-h Ambulatory BP Variables in Patients With AD

The bivariate Spearman correlation was used to analyze the correlations between the levels of pathological biomarkers of AD in CSF and 24-h ambulatory BP variables in patients with AD (Table 6). The results showed that the levels of Aβ_1−42_, P-tau, and T-tau in CSF were not correlated with 24-h BP value, CV of BP, and pulse pressure in patients with AD.

**Table 6:** Correlations between neuropathological biomarkers of AD and 24-h ambulatory BP variables in patients with AD.

	**A β_1−42_**		**P-tau**		**T-tau**	
	***R***	***P-*value**	***R***	***P-*value**	***R***	***P-*value**
Average 24-h SBP value	−0.031	0.843	0.014	0.930	−0.024	0.879
Average daytime SBP value	−0.018	0.910	0.050	0.749	−0.005	0.975
Average night-time SBP value	−0.055	0.728	−0.058	0.714	−0.125	0.424
Average 24-h DBP value	−0.242	0.118	−0.086	0.585	−0.087	0.578
Average daytime DBP value	−0.233	0.132	−0.036	0.817	−0.094	0.549
Average night-time DBP value	−0.196	0.209	−0.188	0.229	−0.076	0.627
24-h CV of SBP	0.114	0.467	0.071	0.649	0.050	0.750
Daytime CV of SBP	0.039	0.805	0.013	0.933	−0.014	0.927
Night-time CV of SBP	0.091	0.563	0.043	0.784	0.138	0.378
24-h CV of DBP	0.073	0.644	−0.007	0.964	−0.010	0.948
Daytime CV of DBP	0.037	0.814	−0.031	0.846	−0.016	0.920
Night-time CV of DBP	0.042	0.790	−0.084	0.594	−0.094	0.551
24-h ambulatory pulse pressure	0.224	0.149	0.164	0.293	0.064	0.683
Daytime ambulatory pulse pressure	0.206	0.186	0.181	0.244	0.063	0.686
Night-time ambulatory pulse pressure	0.175	0.260	0.057	0.714	−0.029	0.856

*AD, Alzheimer's disease; Aβ, β amyloid; T-tau, total tau; P-tau, phosphorylated tau; SBP, systolic blood pressure; DBP, diastolic blood pressure; CV, coefficient of variation*.

## Discussion

AD is one of the most common age-related disorders, and the likelihood of AD progression increased with age (Lai et al., 2020). In this study, it was found that the age of the AD-D group was significantly higher than that of the AD-MCI group, which was in line with the progressive process of AD. A previous study revealed that the memory of patients with AD decreased linearly with age (Messinis et al., 2016). For patients with AD, in addition to memory, language and attention were also the cognitive domains prone to functional decline with age (Blenkinsop et al., 2020). In this study, we found that the increase in age was the independent influencing factor of the impairments of memory, language, and attention in patients with AD. Age remained the greatest risk factor for AD, and was thus a fundamental driver for the development and progression of the disease (Masters, 2020).

In this study, the median course of disease in the AD-D group (36.00 months) was longer than that in the AD-MCI group (24.00 months), with *p*-value was close to 0.05, demonstrating that AD was a continuous process, and MCI was the early stage of the disease (Bondi et al., 2017). It was previously found that language dysfunction happened at the primary stage of AD and developed over time (Khatoonabadi and Masumi, 2019). As the disease progressed from MCI to dementia, a continuous decline in language was observed in patients with AD (Szatloczki et al., 2015). Here, we found that the increased course of disease was one of the independent factors affecting language in patients with AD.

Increasing data suggested that BPV was related to cognitive impairment. It was found that the rate of cognitive decline in patients with AD with high BPV was significantly higher than in those with low BPV (de Heus et al., 2019). The possible mechanisms underlying increased BPV and the severity of AD are currently unclear. It was speculated that increased BPV might induce variability of cerebral perfusion and impact brain health and cognition (Sible and Nation, 2020). Another possible explanation was that arteriosclerosis might contribute to BPV inflation and AD-related cognitive decline (Ma et al., 2020). Arterial stiffness increased BPV through different mechanisms and affected cognitive function (Hughes et al., 2018). Alternatively, the effects of neurodegeneration on the cortical control of the autonomic nervous system might cause the amplification of BPV (Nagai et al., 2010). AD pathology affecting central nervous system control of autonomic activity might influence BP and BPV (Sturm et al., 2018; Betts et al., 2019). A previous systematic review and meta-analysis showed that systolic BPV was significantly associated with a deterioration in cognitive impairment, and diastolic effect sizes were less stronger than systolic effect sizes in a direct comparison, including both BPV and mean BP for cognitive impairment or dementia (de Heus et al., 2021). This study found that the variability of daytime SBP in the AD-D group was significantly higher than that in the AD-MCI group. Considering that the increased daytime SBP variability was related to the severity of AD, it might be an effective predictor of disease progression for patients with AD. In a previous report, it was observed that increased systolic BPV was associated with arterial stiffening, whereas diastolic BPV was not (Zhou et al., 2018), which might be one of the reasons why systolic BPV had a greater impact on cognitive function than diastolic BPV.

Language dysfunction was well correlated with the impairment of temporal lobe (Chang et al., 2017). A previous study found that the elevated BPV was related to medial temporal volume loss specifically in those with abnormal AD biomarkers (Sible and Nation, 2021a). This study found that the increase in daytime SBP variability was an independent factor affecting the language function in patients with AD. Thus, the rate of language decline in patients with AD may be decreased by reducing daytime SBP variability. In the process of BP management of patients with AD, we must pay attention to the management of BPV, especially daytime SBP variability.

BP level was closely related to the occurrence of AD. Mid-life hypertension was associated with an increased risk of AD, while elevated late-life BP might be related to a decreased risk of AD (Ou et al., 2020). The role of BP level on the progression of AD was unclear. In this study, there was no significant difference in the average 24-h, daytime, and night-time SBP and DBP between the AD-MCI and AD-D groups. Thus, the relationship between BP level and severity of AD might not be a simple linear relationship. High pulse pressure was correlated with cerebral microvascular damage as well as white matter structural differences in elderly patient brains (Levin et al., 2020). This study found that the BP level and pulse pressure had certain correlations with the scores of memory, language, and attention in patients with AD. However, further regression analysis did not show that BP level and pulse pressure were the independent factors affecting memory, language, and attention of patients with AD, suggesting that BP level and pulse pressure might be related to cognitive impairment of patients with AD by affecting other factors. The above data indicated that the effect of BPV on cognitive function in patients with AD was greater than that of BP level and pulse pressure. The value of BPV in predicting cognitive decline and dementia risk may be beyond that of the average BP level and pulse pressure.

Pathological biomarkers of AD mainly contained Aβ_1−42_ and P-tau, which depositions led to neuronal damage by a series of pathways, and then induced memory decline and cognitive impairment (Xin et al., 2018). Aβ_1−42_ in CSF was widely accepted as a biomarker for AD, but histopathological evidence suggested that the Aβ_1−42_ level was a relatively weak predictor of severity of cognitive impairment compared with P-tau (Chandra et al., 2019). T-tau in CSF was a common biomarker for neurodegeneration with high sensitivity, but not specific for AD (Rabbito et al., 2020). In this study, the T-tau level in CSF from the AD-D group was significantly lower than that in the AD-MCI group, indicating that T-tau level in CSF was also one of the better indicators predicting the degree of neurodegeneration and progression of AD. Previous studies have explored the potential mechanism of cognitive impairment caused by the increased BPV. In this study, we found that the levels of Aβ_1−42_, P-tau, and T-tau in CSF were not significantly correlated with 24-h BP level, CV of BP, and pulse pressure in patients with AD. It suggested that 24-h ambulatory BP variables might not affect the cognitive function of patients with AD by changing the levels of Aβ_1−42_, P-tau, and T-tau in CSF. Neurodegeneration in the AD-D patients was significantly more severe than that in the AD-MCI. However, the association between 24-h BP variables and pathological biomarkers of AD was weak.

Certain limitations existed in this study are as follows: first of all, CSF samples were not obtained from all patients with AD enrolled in this study due to the difficulties caused by multiple reasons, which might decrease the statistical power of the analyses. In addition, as an observational study, this study provided limited grounds for drawing definite conclusions, and longitudinal studies are required to further clarify the impact of 24-h ambulatory BP variables on the progression and prognosis of patients with AD.

In summary, patients with AD in the dementia stage had significantly increased daytime CV of SBP than those in the MCI stage. Multiple 24-h ambulatory BP variables were correlated with cognitive domains of memory, language, and attention, and the daytime CV of SBP was the independent influencing factor of language. Patients with AD in the dementia stage had significantly severe neurodegeneration than those in the MCI stage, which was, however, not based on the influence of 24-h ambulatory BP variables on neuropathological biomarkers of AD.

## Data Availability Statement

The original contributions presented in the study are included in the article/supplementary material, further inquiries can be directed to the corresponding author/s.

## Ethics Statement

The studies involving human participants were reviewed and approved by the Ethical Review Board of Beijing Tiantan Hospital. The patients/participants provided their written informed consent to participate in this study.

## Author Contributions

LL and WeiZ designed the study and revised the manuscript. LL, PG, TL, WeijiaoZ, MH, JL, HG, DL, and WeijiaZ recorded the clinical data. LL, WW, and WeiZ carried out data analysis and wrote the manuscript. LL, WW, PG, and TL suggested the important data analysis. All authors contributed to the article and approved the submitted version.

## Funding

This research was funded by the National Key Research and Development Program of China (2016YFC1306300 and 2016YFC1306000); the National Key R&D Program of China-European Commission Horizon 2020 (2017YFE0118800-779238); the National Natural Science Foundation of China (81970992, 81571229, 81071015, and 30770745); Capital's Funds for Health Improvement and Research (CFH) (2022-2-2048); the Key Technology R&D Program of Beijing Municipal Education Commission (kz201610025030); the Key Project of Natural Science Foundation of Beijing, China (4161004); the Natural Science Foundation of Beijing, China (7082032); Project of Scientific and Technological Development of Traditional Chinese Medicine in Beijing (JJ2018-48); Capital Clinical Characteristic Application Research (Z121107001012161); High Level Technical Personnel Training Project of Beijing Health System, China (2009-3-26); Project of Beijing Institute for Brain Disorders (BIBD-PXM2013_014226_07_000084); Excellent Personnel Training Project of Beijing, China (20071D0300400076); Important National Science and Technology Specific Projects (2011ZX09102-003-01); National Key Technology Research and Development Program of the Ministry of Science and Technology of China (2013BAI09B03); Project of Construction of Innovative Teams and Teacher Career Development for Universities and Colleges Under Beijing Municipality (IDHT20140514); Beijing Healthcare Research Project, China (JING-15-2); Basic-Clinical Research Cooperation Funding of Capital Medical University, China (2015-JL-PT-X04, 10JL49, and 14JL15); Natural Science Foundation of Capital Medical University, Beijing, China (PYZ2018077); Youth Research Funding, Beijing Tiantan Hospital, Capital Medical University, China (2015-YQN-14, 2015-YQN-15, and 2015-YQN-17).

## Conflict of Interest

The authors declare that the research was conducted in the absence of any commercial or financial relationships that could be construed as a potential conflict of interest. The handling editor JL declared a shared parent affiliation with the authors at the time of review.

## Publisher's Note

All claims expressed in this article are solely those of the authors and do not necessarily represent those of their affiliated organizations, or those of the publisher, the editors and the reviewers. Any product that may be evaluated in this article, or claim that may be made by its manufacturer, is not guaranteed or endorsed by the publisher.
